# Beginning of a journey: unraveling the mystery of chronic kidney disease of unknown aetiology (CKDu) in Sri Lanka

**DOI:** 10.1186/s12992-017-0268-y

**Published:** 2017-06-30

**Authors:** Jacob Kumaresan, Ruwanika Seneviratne

**Affiliations:** 1 World Health Organization Country Representative to Sri Lanka, No 05, Anderson Road, Colombo 05, Sri Lanka; 2 Ministry of Health, Nutrition and Indigenous Medicine, No 385, Rev Baddegama Wimalawansa Thero Mawatha, Colombo 10, Sri Lanka

**Keywords:** CKDu, Research, Surveillance of chronic kidney disease

## Abstract

**Background:**

Globally, chronic kidney disease of unknown aetiology (CKDu) is observed in several areas and among specific ethnic or occupational groups. Given the widespread environmental pollution and the proportions of agriculture workers world-wide, CKDu may be the next global public health issue demanding attention. Recent escalation of CKDu in Sri Lanka has caused a serious public health crisis in the country, made worse by lack of national data.

**Main text:**

The specific geographic distribution, preponderance among farming population, similar histology findings and absence of usual risk factors for kidney disease indicate undetected nephrotoxic agents playing a role in causation. Some of the challenges for the country are uncoordinated preventive efforts, diverse opinions among stakeholders on causality and fragmented research efforts with limited focus on potential causes of CKDu. As a result, accurate estimation of the CKDu burden, identification of causative agents and implementation of effective actions have been delayed. Stakeholder engagement, with involvement of international experts has been the starting point for finalizing a working case definition to establish community based surveillance as a future platform to conduct long-term research.

**Conclusion:**

The country is now poised to contribute to global knowledge by solving the mystery of ‘u’ in CKDu. This commentary highlights the importance and the mechanisms of making an effective breakthrough as early as possible; failing which CKDu can progress rapidly as demonstrated by the situation in Sri Lanka.

## Background

The escalation of chronic kidney disease of unknown aetiology (CKDu) in Sri Lanka, which emerged in the early 1990s, has caused a serious public health crisis in the country [1]. In Sri Lanka, non-communicable diseases (NCDs) and problems related to old age are rapidly increasing due to the epidemiological and demographic transitions. High life expectancy at birth and prevalence of diabetes and hypertension have resulted in conditions such as Chronic Kidney Disease (CKD). However, the recent emergence of a particular form of CKD with similar histology, in a specific geographic location, commonly within the farming population, in the absence of usual risk factors; indicate the likelihood of one or more yet undetected nephrotoxic environmental agents playing a role in causation [1, 2].

Globally, a similar scenario is observed with rise in CKD ‘hotspots’ either in specific geographical regions or among specific ethnic or occupational groups. Cadmium-related nephropathy in Japan, Balkan endemic nephropathy in countries around the Danube river basin, Uddanam endemic nephropathy in India and Mesoamerican nephropathy among sugar cane cutters are some of the key endemic nephropathies reported in literature [3, 4] . In some areas, the causes have been identified whereas in other areas including Sri Lanka, the aetiology remains unknown.

The current disease surveillance system of the country does not provide accurate estimation of the disease burden on a national scale. Due to the lack of national registries and limited reporting of non-communicable diseases, the prevalence of CKDu in Sri Lanka is not known [5]. A number of studies report prevalence for geographical regions. As the study populations and case definitions vary widely caution needs to be exercised in interpreting findings.

Although several aetiological studies, including laboratory, environmental and population studies have been conducted in Sri Lanka, there are many gaps in literature due to limited focus on potential risk factors and inadequate samples [5–7].

Primary prevention, early diagnosis and multi-stakeholder engagement for action are some cost-effective options available for a low-middle income country like Sri Lanka to tackle this problem. However, with different views being held by the scientific communities in the country, opinions remain divided. Health authorities are now attempting to streamline research activities and establish surveillance of CKDu with the involvement of all relevant stakeholders. The objective of this article is to present how, as a developing low-middle income country with restricted resources, Sri Lanka is addressing this unique challenge.

### Endemic nephropathy in Sri Lanka- CKDu

Sri Lanka, with a population of over 20 million has commendable health services with indices on par with developed countries, achieved through comparatively low resources [8]. This success has been attributed mainly to free healthcare and education policies since 1920s. The economic burden due to CKDu is significant with 4.6% of the Annual Health Budget in 2010 [9] being spent on managing patients. The free public health care service struggles to cater to the rising demand of chronic ailments including renal replacement therapy. With over 70,000 estimated patients with renal diseases, this is a national priority needing urgent attention [2].

Chronic kidney disease of unknown etiology (CKDu) is CKD without diabetes, hypertension, preexisting renal conditions, or snake bite. It is slowly progressive, irreversible, and asymptomatic until late stages [2]. In Sri Lanka, CKDu is observed more commonly in the North Central Province (NCP), of the dry zone, located in the fluoride belt: it is populated with farming communities which rely heavily on agrochemicals [1, 6]. CKDu primarily affects paddy farmers living in these areas [1, 7, 10], the exact cause/s of which is still unclear despite more than 20 years of research. A population prevalence study conducted among 4957 participants from three endemic districts of NCP in 2010 and 2011 showed an age-standardized prevalence of 12.9% (95% confidence interval [CI]; 11.5%–14.4%) in males and 16.9% (95% CI; 15.5%–18.3%) in females. Males (23.2%) had the more severe form of the disease compared to females (7.4%). Patients range in age from 17–70 but are most commonly men aged 30–60 [2].

In another study, population surveys were carried out in three different provinces in Sri Lanka. Medawachchiya, located in NCP; Yatinuwara, located in the Central Province (CP); and Hambanthota in the Southern Province (SP) were sampled (*n* = 6153, 87% response rate) [5]. In the regions selected in the NCP, 84% had patients with CKDu, compared with CP which reported 2.9% and SP which had 9.1% [5].

In Sri Lanka, histo-pathological samples demonstrate tubulo-interstitial disease which indicate toxic nephropathy pointing to environmental agents playing a role in pathogenesis [2, 5, 10]. Factors implicated are pesticides such as organophosphates; Hantavirus; fungal toxins; alcohol consumption; heavy metals due to agrochemical use; dry, humid climate and hard water consumption. It is postulated that several agents and genetic predisposition may play a role in causation [1, 2, 10].

The country faces many challenges due to the ‘epidemic’ of CKDu. Continuing the provision of free-healthcare services to those with CKDu is a major concern. The burden due to CKDu continues to increase and, in the absence of knowledge on causative agents the country has not yet been able to make tangible progress in the prevention and control of CKDu. Challenges are identifying clear uniform criteria for population screening, providing long term care free-of-charge and preventing the younger generations from exposure to the possible causative agents. In order to overcome these challenges health system strengthening, and reforms to address the growing burden of non-communicable diseases have been identified. This includes financing and revisiting the roles and responsibilities of the health workforce in the country. With this background, the need for a systematic national approach to address CKDu becomes an important turning point in the planned health reforms.

### National approaches to addressing CKDu

The starting point for addressing CKDu was identifying and setting up a multi stakeholder group focused on advancing prevention and control of CKDu. To provide oversight and coordinate efforts, a Presidential Task Force (PTF) on CKDu was set up in 2014 bringing much needed political backing and funding for CKDu from the national budget.

In 2016, WHO and PTF convened an international expert consultation, with national and international experts, to guide future direction for addressing CKDu in Sri Lanka. Development of a robust surveillance system; conduction of interdisciplinary research; strengthening implementation of interventions; provision of social support to patients, family and community; and, developing a framework for monitoring and accountability were key recommendations from this consultation.

A holistic National Action Plan 2016–2020 was developed by PTF based on recommendations of the international consultation, with an overall goal of curbing the epidemic by understanding the aetiology of the problem. Interim targets are provision of safe water to alleviate suffering of affected persons and their families; education on prevention of illness/progression of disease; inter-sectoral collaboration among various partners on intervention strategies; multifaceted research to determine causal factors; and, targeted communication to the communities. The ministries of Health; Agriculture and Social Empowerment & Welfare will be the key partners involved in implementation of specific tasks. In addition, National Water Supply & Drainage Board and the ministries of Finance; Education; Mass media; and Academia, along with civil societies and United Nations (UN) partners will be involved.

A standardized, uniform case definition was also developed with the consensus of nephrologists, physicians, clinical and non-medical researchers, epidemiologists, programme managers at national and district levels. The Ministry of Health has now embarked on community-based surveillance with the intention of generating national data and for conducting long-term research on potential causative factors. Funding sources are the Government and the National Science Foundation.

## Conclusions

Epidemiological and demographic transitions favouring a rise in NCDs, along with high usage of agrochemicals and widespread environmental pollution can cause conditions such as CKDu, a threat which many countries may not be immune to. Given the widespread environmental pollution and the high proportions of global communities engaged in agriculture, CKDu may be the next global public health problem demanding attention. In Sri Lanka, the stage is now set to better understand the burden, geographical distribution and trends of CKDu and the country is on the road to solve the mystery of ‘u’ in CKDu and contribute to the global knowledge on CKDu.

This commentary highlights the importance of making an effective breakthrough as early as possible; failing which conditions such as CKDu can progress rapidly as demonstrated by the situation in Sri Lanka.

In parallel to understanding the aetiology, provision of universal access to preventive, diagnostic and curative healthcare services will stem the tide. Considering the rising burden due to CKDu and other non-communicable diseases, a reform of the health services toward promotion of wellbeing and prevention of ill health in populations is necessary to reduce the burden in the long run. Adoption of a more coordinated approach at national level, engaging all stakeholders and involving the global scientific community will cutback limited resources spent on fragmented and futile efforts and ultimately save lives.

## Abbreviations

CKDu: Chronic kidney disease of unknown aetiology
NCD: Non-communicable diseases
NCP: North Central Province
PTF: Presidential Task Force
WHO: World Health Organization
